# The Hamstrings: Anatomic and Physiologic Variations and Their Potential Relationships With Injury Risk

**DOI:** 10.3389/fphys.2021.694604

**Published:** 2021-07-07

**Authors:** José Afonso, Sílvia Rocha-Rodrigues, Filipe M. Clemente, Michele Aquino, Pantelis T. Nikolaidis, Hugo Sarmento, Alberto Fílter, Jesús Olivares-Jabalera, Rodrigo Ramirez-Campillo

**Affiliations:** ^1^Centre for Research, Education, Innovation and Intervention in Sport, Faculty of Sport of the University of Porto, Porto, Portugal; ^2^Escola Superior de Desporto e Lazer, Instituto Politécnico de Viana do Castelo, Viana do Castelo, Portugal; ^3^Research Centre in Sports Sciences, Health Sciences and Human Development, Vila Real, Portugal; ^4^Tumor & Microenvironment Interactions Group, Instituto de Investigação e Inovação em Saúde, Porto, Portugal; ^5^Instituto de Telecomunicações, Delegação da Covilhã, Covilhã, Portugal; ^6^Department of Health and Sport Sciences, Adelphi University, New York, NY, United States; ^7^School of Health and Caring Sciences, University of West Attica, Athens, Greece; ^8^Research Unit for Sport and Physical Activity, Faculty of Sport Sciences and Physical Education, University of Coimbra, Coimbra, Portugal; ^9^FSI Sport Research Lab, Football Science Institute, Granada, Spain; ^10^Research Group Physical Activity, Health and Sport CTS-948, University of Pablo de Olavide, Seville, Spain; ^11^Sport and Health University Research Institute, Department of Physical and Sports Education, University of Granada, Granada, Spain; ^12^Department of Physical Activity Sciences, Universidad de Los Lagos, Santiago, Chile; ^13^Centro de Investigación en Fisiología del Ejercicio, Facultad de Ciencias, Universidad Mayor, Santiago, Chile

**Keywords:** hamstrings injuries, interindividual variation, kinesiology, muscle architecture, exercise prescription, hamstrings anatomy

## Abstract

The incidence and recurrence of hamstrings injuries are very high in sports, posing elevated performance and financial-related costs. Attempts to identify the risk factors involved in predicting vulnerability to hamstrings injury is important for designing exercise-based programs that aim to mitigate the rate and severity of hamstrings injuries and improve rehabilitation strategies. However, research has shown that non-modifiable risk factors may play a greater role than modifiable risk factors. Recognizing non-modifiable risk factors and understanding their implications will afford the prescription of better suited exercise programs, i.e., that are more respectful of the individual characteristics. In a nutshell, non-modifiable risk factors can still be acted upon, even if indirectly. In this context, an underexplored topic is how intra and inter- individual anatomic and physiologic variations in hamstrings (e.g., muscle bellies, fiber types, tendon length, aponeurosis width, attachment sites, sex- and age-related differences) concur to alter hamstrings injuries risk. Some anatomic and physiologic variations may be modifiable through exercise interventions (e.g., cross-sectional area), while others may not (e.g., supernumerary muscle bellies). This apparent dichotomy may hide a greater complexity, i.e., there may be risk factors that are partially modifiable. Therefore, we explored the available information on the anatomic variations of the hamstrings, providing a deeper insight into the individual risk factors for hamstrings injuries and contributing with better knowledge and potential applications toward a more individualized exercise prescription.

## Introduction

The hamstrings are a hot topic in Sports Sciences, with a PubMed search using the terms “hamstring^∗^” and “sport^∗^” from the year 2020 to the present showing >500 entries. This is because hamstrings injuries are common in the athletic community (Monajati et al., 2016; Sheean et al., 2021). While hamstrings strain injury (HSI) is a commonly used term, it does not encompass all hamstrings injuries; therefore, HSI will only be used if the cited authors specifically refer to that subset of hamstrings injuries. The number of non-contact training-related hamstrings injuries in different sports have increased gradually and systematically [e.g., an annual average 2.3% increase in the total hamstrings injuries rate over the 13-year period (2001–2014)] (Ekstrand et al., 2016; Ertelt and Gronwald, 2017). Anatomic and functional aspects of the hamstrings, including the fact that muscles cross two joints (except the short head of the biceps femoris) and that eccentric action during running or stretching carried out to extreme joint positions, make it prone to vulnerable to strain-related injuries (Edouard et al., 2016).

A systematic review including 13 studies with more than 3,800 athletes and two million sport exposure hours reported an incidence of acute hamstrings injuries ranging from 0.3 to 0.5 per 1,000 exposure hours in women, to 0.3–1.9 per 1,000 exposure hours in men, accounting for 5% to 15% of all soccer-related injuries, with recurrence rates varying from 4 to 68% (Diemer et al., 2021). The prevalence of hamstrings injuries were reported to be more than 60% (unilateral) and more than 30% (bilateral) in baseball players, with ∼30% recurrence rates (Zachazewski et al., 2019), while prevalence rates of 40% were reported for professional soccer players (Ribeiro-Alvares et al., 2020). This is challenging in terms of recovery time and financial costs (Pickering and Kiely, 2018; Shield and Bourne, 2018; Metcalf et al., 2019). In the Australian Football League, data from 10 competitive seasons determined that the financial cost of HSI per club increased by 71% from 2003 to 2012 (Hickey et al., 2014).

A wide body of research explored the implementation of specific exercise-based programs for mitigating hamstrings injury risk (Al Attar et al., 2017; Chebbi et al., 2020). Due to intra- and inter-muscular differences in this group of muscles, exercises designed for the hamstrings may not provide equal stimuli for semitendinosus (ST), semimembranosus (SM), and biceps femoris (BF) (Kellis, 2018). Relevant works suggested that non-modifiable factors (i.e., older age, previous injury) may have a greater impact on risk for hamstrings injuries compared to modifiable ones (Freckleton and Pizzari, 2013; Green et al., 2020). Non-modifiable factors could also extend to aspects of human anatomy which are not possible to modify, such as variations in insertions sites and the presence of supernumerary muscle bellies (Tubbs et al., 2016).

A better knowledge of interindividual variation in hamstrings anatomy and geometry could allow the design of better-individualized interventions, eventually reducing the likelihood of suffering (and aid in the recovery from) an injury (Ertelt and Gronwald, 2017). Interindividual variation in strength levels (specifically eccentric strength), hamstrings muscle fascicle length, muscle-tendon architecture, muscle fiber type and region-specific innervation have been hypothesized as relevant factors for risk of HSI (Timmins, 2017; Huygaerts et al., 2021), and genetic variations have been explored in response to loading and post-exercise recovery (Pickering and Kiely, 2018). However, anatomic and physiologic variations of the hamstrings are more diversified than highlighted here (Tubbs et al., 2016).

Our goal was to explore the available information on the anatomic variations of the hamstrings, provide a deeper insight into the individual risk factors for hamstrings injuries and contribute evidence-based applications for more individualized intervention regimens. Although acute injury prediction, given its multifactorial nature, will probably never be possible (Bahr, 2016), some risk factors can be controlled for. In the first part of this commentary, an overview of hamstrings injuries is provided, including injury mechanisms, both non- and modifiable risk factors, exercise-based strategies to mitigate risk factors, as well as topics of rehabilitation and return to play (RTP). While the literature often dichotomizes risk factors into modifiable and non-modifiable, some non-modifiable risk factors may be partially modifiable, as will be discussed. In the second part, “normal” anatomy of the hamstrings is described, and the inter- and intraindividual variations of hamstrings anatomy and how they might be related to injury risk are explored, considering modifiable and non-modifiable anatomic variations and how to recognize them to prescribe better adjusted exercise. Non-modifiable risk factors can still be acted upon, by designing exercise interventions that are more respectful of those factors.

A systematic review of the literature was not performed, given the scope and goals of this commentary. However, we selected literature published in peer-reviewed journals, most of which indexed in PubMed. The goal was to gather relevant information on a wide variety of topics related to our theme. Where controversy existed, we attempted to provide a balanced discussion, selecting studies with contradictory findings. Where appropriate, renowned textbooks were used (Standring, 2015; Tubbs et al., 2016).

## Part 1: An Overview of Hamstrings Injuries

### Hamstrings Functions and Injury Mechanisms

The hamstrings are involved in major movements of daily life, such as standing and walking. In the standing position, the line of gravity passes behind the hip, producing an extension moment that needs to be counterbalanced by the hip flexors (Houglum and Bertoti, 2012). On the contrary, the hamstrings need to act toward knee flexion, since the line of gravity falls in front of the knee inducing hyperextension (Smith, 1957). During walking, the hamstrings (and gluteus maximus) act on the hip during the final leg swing phase by generating an eccentric action to decelerate the rate of hip flexion, and concentrically in the following hip extension (Hogervorst and Vereecke, 2015). During the leg swing phase, the hamstrings act eccentrically to decelerate the rate of knee extension (Houglum and Bertoti, 2012).

The hamstrings are important for sport activities involving sprints, jumps, tackles, cutting maneuvers and kicking (Ekstrand et al., 2012). The motion of the kicking leg (e.g., soccer kick) includes a backward and forward motion (Lees and Nolan, 1998). During the backward motion, the hamstrings act using concentric action in hip extension (together with gluteus maximus) and knee flexion and lateral rotation (only the BF) (Fields et al., 2005). During the forward motion, the hamstrings act eccentrically to decelerate hip and knee during respective hip flexion and knee extension motions (Fields et al., 2005). The decelerating action of hamstrings occur at a position (hip flexion and knee extension) where these muscles are passively insufficient (i.e., the muscles reach their maximal length since they are biarticular muscles passing through hip and knee) (Coole and Gieck, 1987). For these reasons, the hamstrings are prone to injury during high-velocity running or sprinting, due to eccentric over-loading at the end of the swing phase (Jönhagen et al., 1994).

A systematic review with 26 studies explored the mechanisms behind the hamstrings injuries (Danielsson et al., 2020); the authors stratified the mechanisms according to the methods used to determine the injury: (i) stretch-related injuries; (ii) kinematic analysis; (iii) electromyography-based kinematic analysis; and (iv) strength-related injuries. In the first group (i.e., stretch-related injuries), all studies reported that hamstrings injuries occurred upon extensive hip flexion with hyperextension of the knee (i.e., the hamstrings were strongly stretched at both joints it crosses, which are the hip and knee). In the review (Danielsson et al., 2020), hamstrings injuries were associated with running-based actions and especially to the late swing phase of the running gait cycle, which may imply a powerful eccentric action. These powerful actions increase as running velocity increases (Higashihara et al., 2010b).

### Modifiable Risk Factors

Modifiable risk factors can be modified under certain interventions. In the case of hamstrings risk factors, these include player load, warm-up preparations, lumbo-pelvic hip stability, motor patterns (e.g., “Groucho” position), cardiovascular fitness, fatigue, mobility, low back pain, recovery strategies, strength, asymmetry, nutrition and psychosocial factors (Liu et al., 2012; Mendiguchia et al., 2012; Schuermans et al., 2014; Buckthorpe et al., 2019; Huygaerts et al., 2020). Using proper exercise-based programs, establishing a balance between load and recovery, carefully structuring of the intervention sessions, and providing adequate physical preparation, can readily tackle most of these risk factors, which is valid for nearly all injuries. We do not aim to provide an in-depth discussion of modifiable risk factors, as it would escape the main goals of our work. However, we feel it is relevant to briefly discuss four topics that should be more widely acknowledged (i.e., fatigue) or seen under a more critical perspective (i.e., warm-up, flexibility and functional asymmetry).

#### Fatigue

Fatigue was associated with decreased eccentric hamstrings strength and an altered neuromuscular coordination, suggesting a higher risk of developing an injury (Jones et al., 2015; Buckthorpe et al., 2019; Huygaerts et al., 2020). In the review of Danielsson et al. (2020), fatigue was associated to HSI, underlining that, aside from strength and flexibility, endurance was a relevant risk factor (Watson et al., 2017; Farley et al., 2020). Eccentric strength endurance of the hamstrings was reported to be significantly lower in male soccer players with previous hamstrings injuries (Schuermans et al., 2014). A study with elite footballers (*n* = 50) presented contradictory findings when comparing the previously injured limbs (*n* = 11) with non-injured limbs (*n* = 89) (Freitas et al., 2021), but the athletes acquired hamstrings injury in the previous 2 years, possibly providing enough time and exercise intervention for the hamstrings to recover their endurance levels. Previous injuries are non-modifiable risk factors, but improvements in endurance and increased tolerance to fatigue can be achieved with exercise training (Delextrat et al., 2018). In this sense, both detraining and hamstrings damage (secondary to a previous injury) may be considered partially modifiable factors.

#### The Warm-Up

The role of warm-up in reducing injury risk is not clear (Fradkin et al., 2006; McCrary et al., 2015), and adherence to warm-up protocols may interfere with its effectiveness (Owoeye et al., 2020), i.e., the frequency and degree of compliance to the warm-up protocol may largely dictate how effective the protocol is. It is unclear whether different types of warm-ups induce distinct acute effects on modifiable injury risk factors (Niederer et al., 2020). A prospective two-season study registered posterior thigh injuries in 83 Australian rules football players, of which 62 were confirmed to have hamstrings injury through magnetic resonance imaging (MRI) (Verrall et al., 2003). In this study, 15% of hamstrings injuries occurred during the warm-up period and 85% after the warm-up (Verrall et al., 2003). Considering that the main part of an exercise session is longer than the warm-up, these percentages of distribution provide insufficient information. Harking back to the discussion surrounding fatigue (Delextrat et al., 2018; Danielsson et al., 2020), it could be speculated that overly long and/or intense warm-ups may generate excessive fatigue for the remainder of the training session. Warm-up protocols aiming to produce performance potentiation usually generate potentiation and fatigue, and this balance varies from individual to individual (Afonso et al., 2019; Blazevich and Babault, 2019). Regarding hamstrings injury prevention, individually monitoring responses to warm-up is suggested.

#### The Role of Flexibility

A clear relationship between hamstrings flexibility and injury risk has also not been established (Worrell and Perrin, 1992; Christopher et al., 2019). de la Motte et al. (2019) assessed 27 studies and explicitly stated they considered studies showing *association* between flexibility and other factors and musculoskeletal injury in military and civilian populations. The authors showed there was moderate yet conflicting evidence associating hamstrings flexibility and musculoskeletal injury risk. It is easy for readers to interpret this association using a causative framework, but caution should be used, as previous hamstrings injuries reduce flexibility until 20–30 days post-injury (Maniar et al., 2016), but reduced flexibility does not necessarily increase injury risk. In this context, the role of stretching in reducing injury risk is controversial (Herbert and Gabriel, 2002; Small et al., 2008; Behm et al., 2015), specifically in the case of hamstrings injuries (Liu et al., 2012; Rogan et al., 2013).

#### Functional Asymmetry

Some degree of interlimb asymmetries in hamstrings strength is the norm (Boccia et al., 2018; Kulas et al., 2018; Cuthbert et al., 2021) and, if not excessive, are beneficial for performance of tasks involving change of direction and sprinting, without impaired jumping (Coratella et al., 2018). Injured soccer players exhibit a more symmetrical recruitment pattern between ST, SM and BF (corresponding to a less economic hamstrings muscle activation), as opposed to the more asymmetric pattern of subjects without previous injuries (Schuermans et al., 2014). Athletes with previous hamstrings injuries also demonstrated a decrease of ST metabolic activity, compensated by a greater involvement of the BF (Schuermans et al., 2014). The mechanisms underlying the symmetrical muscle activation possibly involve a compensatory increase of BF activation and a maladaptation neuromuscular coordination behavior, resulting in a less efficient hamstrings contraction. This condition, combined with peripheral metabolic changes (e.g., earlier onset of pH changes), leads to muscle fatigue and may explain the reduced endurance capacity, associated to a re-injury of hamstrings (Allen et al., 2008; Schuermans et al., 2016; Suarez-Arrones et al., 2021).

### Exercise-Based Strategies for Mitigating Injury Risk

Studies assessing the effectiveness of exercise-based programs in mitigating hamstrings injury risk proliferated. The focus has relied on injury prevention, although we prefer terms such as “risk mitigation,” since there is always an inherent injury risk. A systematic review with meta-analysis (SRMA) including 17 studies showed that exercise-based interventions reduced hamstrings injury risk in ∼50% (Vatovec et al., 2020). However, the quality of assessment through the PEDro (Physiotherapy Evidence Database) scale showed major problems in many studies, with nine having a classification ≤4 (i.e., low methodological quality), and none presenting a classification above six points in the PEDro scale (i.e., no study with high quality). Another SRMA assessed 15 articles studying the effects of including the Nordic Hamstrings exercise (NHE) into wider exercise-based programs in hamstrings injury rate (van Dyk et al., 2019), which resulted in ∼50% risk reduction, but the authors showed that half of analyzed articles had high risk of bias in randomization and allocation concealment, and ∼80% had high risk of bias in blinding of outcome assessments, raising concerns regarding the trustworthiness of the findings. In their inclusion criteria (van Dyk et al., 2019), the intervention could be solely focused on the NHE *or* any wider program including the NHE compared to usual training or alternative programs; it is unclear whether the effects were attributable to the NHE, or to the wider program in which it was included.

Similar results were reported in another SRMA concerning the NHE (Al Attar et al., 2017), although a reasonable risk of bias has been reported elsewhere (Gérard et al., 2020). These two SRMA (Al Attar et al., 2017; Gérard et al., 2020) were alluding to *relative risks*, reducing the trustworthiness on the potential beneficial effect of the NHE on injury prevention. The feasibility of implementing certain protocols into real-world contexts is questionable since the evidence-based interventions are not always effective, i.e., translating into practical applications may be problematic (McCall et al., 2020). Beyond the intrinsic characteristics of each exercise-based program, its effectiveness depends on the buy-in, i.e., how well participants adhere to, and comply with the program (Buckthorpe et al., 2019). Therefore, the belief of coaches and athletes in the programs will interfere with how well they work. Indeed, the placebo and nocebo effects have been observed in sports science (Hurst et al., 2020; Raglin et al., 2020).

Weekly frequency of exercise-based programs to mitigate injury risk is an important parameter: interventions performed ≥2 times per week were more effective in reducing hamstrings injury risk than interventions performed <2 times per week, even though the differences were not large (Vatovec et al., 2020). Previous research suggested that the compliance to the program (i.e., adhering to and performing the sessions) is an important prognostic factor regarding injury risk (Goode et al., 2015; Chebbi et al., 2020).

While prevention programs are biased toward eccentric strength training of the hamstrings, interventions focused on improving endurance (Danielsson et al., 2020), motor control (Ertelt and Gronwald, 2017), lumbo-pelvic dynamics (Shield and Bourne, 2018), using video and technical feedback to improve biomechanical parameters of movement (Monajati et al., 2016), and isometric strength training (Macdonald et al., 2019) should not be neglected. Sprint training is possibly the most common mechanism to simulate the high load and high velocity eccentric actions of the hamstrings (van den Tillaar et al., 2017). A multifactorial, individualized approach should be included in the specific programs aiming to reduce injury risk (Mendiguchia et al., 2012; Lahti et al., 2020; Suarez-Arrones et al., 2021).

### Rehabilitation and Return-to-Play

Rehabilitation and RTP after a hamstrings injury are challenging (Hickey et al., 2017; Taberner et al., 2020). The RTP relies on numerous determinants, such as injury mechanism, level of sport participation, and time to first consultation and pain (Fournier-Farley et al., 2016). It is common for deficits in strength, range of motion (ROM) and muscle morphology alterations to persist after RTP (Sanfilippo et al., 2013; Maniar et al., 2016). Criteria to support safe and appropriate RTP after hamstrings injuries are varied and lack validation (van der Horst et al., 2016). Some exercise-based strategies such as stretching have showed a decreased RTP time, but not reduced the risk of re-injury (Pas et al., 2015), suggesting a misconception in injury management.

The rehabilitation process depends on factors such as the extent and severity of injury, precise anatomic location and the tissue involved (e.g., fascia, muscle and/or tendon). The degree of hamstrings injuries will determine whether surgery is required or not (Ayuob et al., 2020; Sheean et al., 2021), although there is debate regarding the criteria for choosing conservative treatment vs. surgical management (Metcalf et al., 2019; Blakeney, 2020).

### Modifiable Versus Non-modifiable Risk Factors

Despite the potential of exercise-based interventions for mitigating hamstrings injury risk, non-modifiable risk factors should be acknowledged, such as age and previous injury (Mendiguchia et al., 2012). A SRMA involving 71,324 athletes from 78 studies analyzed 8,319 HSIs, of which 967 were recurrences (Green et al., 2020). The stronger factors associated with an increased risk of HSI were older age, previous history of HSI, recent HSI, previous calf strain injury, and previous anterior cruciate ligament (ACL) injury, which are non-modifiable factors (Green et al., 2020). In contrast, modifiable risk factors, such as reductions in strength, strength endurance, power, and motor control, were weakly associated to increased risk for his, and flexibility, mobility or ROM were not associated with risk of HSI (Green et al., 2020).

Non-modifiable risk factors for HSI, such as age and previous injury, were consistently associated with an increased risk of injury in a previous SRMA that included 34 articles (Freckleton and Pizzari, 2013). In this SRMA, the only modifiable factor consistently associated with the risk of hamstrings injuries was the quadriceps concentric peak torque. In a prospective study with Australian football league players (*n* = 125), hamstrings injuries were not associated with NHE strength (Smith et al., 2021). In this study (Smith et al., 2021), player age greater than 25 years and having a previous hamstring, groin or calf injury increased the risk for hamstrings injury. Attention has been devoted to the modifiable risk factors, but non-modifiable factors should be further explored, as well as the mechanisms whereby some non-modifiable factors increase injury risk.

Although it is not possible to erase the existence or previous injuries, or change the age of the player, a better understanding of the hamstrings’ architecture, anatomy and mechanisms may help to deliver a better-individualized exercise-based approach. It was previously established that previous injuries impair ROM (Maniar et al., 2016) and endurance (Farley et al., 2020), both of which can be improved through well-designed exercise intervention protocols. While some architectural features might be modifiable [e.g., cross-sectional area (CSA)], others may not (e.g., variations in insertions), and an understanding of such features may afford a better-individualized exercise prescription. Sex- and age-related risk factors will be explored further in part 2, as they are closely linked to anatomic and physiologic variations. Even when certain factors are non-modifiable, the mechanisms that contribute to the increased injury risk could be individually targeted with better exercise-based interventions, to avoid exacerbating predisposing factors.

### Synopsis of the First Part

The hamstrings are highly relevant for major movements of daily life and sports (e.g., standing, walking, sprinting, cutting), and play an especially important role in decelerating knee extension and hip flexion in high-velocity actions, common in athletic scenarios, such as sprinting. Hamstrings are prone to injuries and so it is important to understand the risk factors involved. Exercise-based interventions for mitigating hamstrings injury risk have emphasized the role of eccentrically biased strength training, although lumbo-pelvic dynamics, technical and motor pattern focused work, sprinting, isometric strength training and general strength and endurance training should not be neglected. Weekly frequency and compliance with the interventions constitutes a relevant factor mediating the effectiveness of exercise-based interventions in mitigating injury risk. However, research suggests that non-modifiable risk factors, namely age and previous injuries, may play a more relevant role in hamstrings injury risk than modifiable risk factors. In this context, relevant anatomic variations may alter the risk of hamstrings injury. Acknowledging the relevant anatomic variations may provide relevant information for prescribing exercise interventions, which will be the focus of the second part of this commentary. Box 1 briefly explores hamstrings’ strength evaluation.

**Box 1. Special box – hamstrings’ strength evaluation.**

Measuring muscle strength allows evaluating and comparing muscle function and performance to obtain a pattern of intramuscular synergistic recruitment between the BF, ST, and SM muscles (Mjølsnes et al., 2004; Schuermans et al., 2014; Wiesinger et al., 2020). Improvement of hamstrings strength is an important strategy to mitigate injury incidence and recurrence, and one of the RTP criteria (Maniar et al., 2016). Early identification and management of excessive strength asymmetries and muscular imbalances assist in the elaboration of a more effective intervention plan to counteract a potential injury risk scenario (Schuermans et al., 2014; Wiesinger et al., 2020). The stationary isokinetic dynamometry (IKD) is a suitable system for assessment of torque during concentric and eccentric knee flexor action (Aagaard et al., 1998; van Dyk et al., 2018; Wollin et al., 2018), although its use is scarce compared to the Nordic hamstrings device (NHD) (Mjølsnes et al., 2004). The NHD detects strength deficits or side-to-side imbalances (Timmins et al., 2016), exercise-related strength progress (Delahunt et al., 2016), and may aid predicting recovery time after injury (McCall et al., 2016). However, van Dyk et al. (2018) reported a weak within-subject correlation (*r* = 0.35), demonstrating a systematic bias toward lower strength values with the NHE. Several methodological specificities were not considered when comparing both methods in previous studies, e.g., IKD and NHD tests were performed under different conditions of joint velocity and hip position (Higashihara et al., 2010a; Guex et al., 2016).

To understand these issues, Wiesinger et al. (2020) compared the mechanical output of hamstrings assessed by using both IKD and NHD methods of 25 healthy male athletes in a counterbalanced repeated-measures protocol. Higher total eccentric work, peak torque at greater knee extension angles, greater side-to-side strength difference, and lower eccentric peak torque were observed in IKD compared to NHD, whereas bilateral strength difference was lower in NHD. The electromyographic analysis showed no difference in the activation of BF and ST during IKD and NHD (Wiesinger et al., 2020). These findings suggest that IKD and NHD methods measured distinct hamstrings muscle activation characteristics. Some aspects were difficult to assess, such as intraindividual strength differences, angle of peak torque and side-to-side differences in eccentric knee flexor strength. It should be highlighted that muscles such as the sartorius, gracilis and gastrocnemius also contribute to knee flexion (Standring, 2015).

Surface electromyography (EMG) allow the assessment of muscle activation patterns (Higashihara et al., 2010a). The EMG activity shows that hamstrings muscles have functional differences, as the SM and BF work harder (i.e., greater EMG) during the initial phase of knee flexion, and the ST at deep flexion angles to complement the decrease in EMG of the other two muscles during open kinetic chain exercise (Hirose and Tsuruike, 2018). Miller et al. (2000) found that during isokinetic movements activation, the BF EMG increased with increasing movement velocity, while the ST and SM EMG activation remained constant during six continuous isokinetic knee extension and flexion movements at 60°, 180°, and 300° s^–1^. In opposition, functional MRI evaluates the intramuscular and intermuscular recruitment patterns with a very high spatial accuracy and detects the magnitude of metabolic activity in muscle tissue although with no real-time information about the amount and timing of the underlying muscle activity (Segal, 2007). Using functional MRI, Schuermans et al. (2014) demonstrated that the more symmetrical and less dissociated the hamstrings muscles work together, the higher the physiological changes would be inside the recruited muscle fibers. Thus, more intramuscular variability can be associated with a reduced metabolic turnover and more economic muscle functioning. MRI-based methods have high costs, risk of injury during assessment, lack of portability and long duration of assessment. Rapid, non-invasive alternative measures of concentric and eccentric hamstrings strength are warranted.

An adapted aneroid sphygmomanometer test (Mondin et al., 2018), a rapid and non-invasive tool, was proposed to assess hamstrings strength. In 14 rugby players, Mondin et al. (2018) found an association between the sphygmomanometer derived pressures at 30 and 90° of knee flexion and isokinetic strength measures, suggesting that this method was reliable to assess hamstrings strength, but not to identify strength asymmetries between dominant and non-dominant legs or knee flexors-to-knee extensors ratios. The limitation of knee flexors-to-knee extensors ratios was explained by the inability of aneroid sphygmomanometer test to measure quadriceps strength at 30° knee flexion with consistency, due to many participants exceeding the readings on the sphygmomanometer scale. This method has a great potential, but requires further studies to be useful as a muscle strength assessment and injury risk screening procedure.

## Part 2: Anatomic and Physiologic Variations of the Hamstrings and Potential Implications for Injury Risk

In this second part, we address the main variations or variants of the hamstrings’ anatomy and physiology, which imply that there is a “normal” or “usual” state of affairs. We start by presenting the commonly described anatomic features of the hamstrings, before engaging in an exploration of their variations.

### Mainstream Anatomic Description of the Hamstrings

We overview the basic hamstrings anatomy as described in the 41st Edition of Gray’s Anatomy (Standring, 2015). Throughout this section, the information derives from this source, unless otherwise stated. The hamstrings, or ischiocrural muscles, comprise the muscles of the posterior compartment of the thigh, and include the ST, SM and BF (long head – BFlh; short head – BFsh), which attach proximally to the ischial tuberosity (except the BFsh). The ST, SM and BFlh are biarticular muscles, and act in extending the coxofemoral joint (hip), as well as flexing and rotating (medially and laterally) the knee. Distally, the ST and SM attach to the tibia, while both heads of the BF attach to the fibula. The hamstrings present relevant interindividual variation in length. The hamstrings are innervated by the sciatic nerve, emerging at the level of S1 vertebra, although with interindividual variations.

**FIGURE 1:**
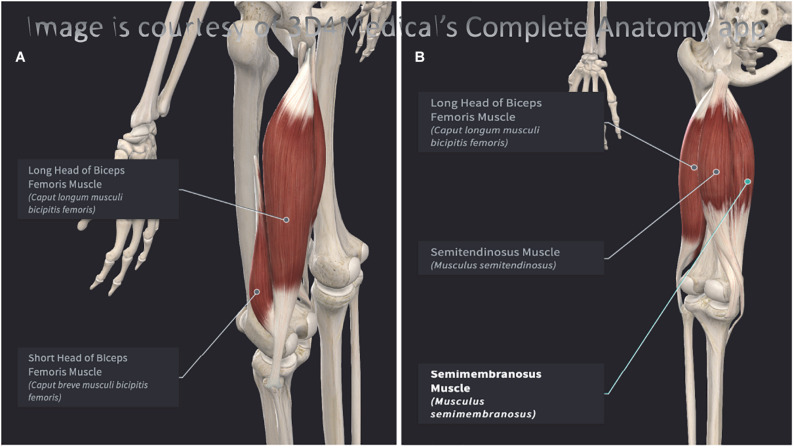
**(A)** Posterolateral view of the left hamstrings. **(B)** Posteromedial view of the left hamstrings. Both images were elaborated with Complete Anatomy 2021, version 7.0.0. (desktop version for Mac OS) and reproduced here with permission (3D4Medical, Elsevier).

Functionally, the hamstrings play an important role in transferring load between the trunk and the lower limbs, and affect the tension of the thoracolumbar fascia. They affect the normal lumbar lordosis, as their length and strength interferes with the position of the innominate bone. When raising the trunk, the hamstrings act in conjunction with the gluteus maximus. It is highlighted that the hamstrings strongly contract in actions involving the need to extend or control the rate of flexion at the hip, while the gluteus maximus contracts when powerful extension of the hip joint is required. The adductor magnus also performs hip extension, has attachments to the ischial tuberosity, and the ischial portion of the adductor magnus shares innervation from the tibial division of the sciatic nerve. The adductor magnus can be considered to have a proper hamstrings portion (Broski et al., 2016; Jeno and Schindler, 2020). Pathology or diminished functional capacity of the adductor magnus and/or the gluteus maximus may result in a greater demand upon the hamstrings. The coordination of the hamstrings with other muscles of the lumbopelvic region may be relevant in understanding hamstrings injuries (Thelen et al., 2006; Shield and Bourne, 2018). Figure 1 presents an overview of the hamstrings muscles.

#### Semitendinosus

The ST has a posteromedial position in the thigh and has a long tendon. Proximally, its tendon is shared with the BFlh, but then these muscles diverge. In the first ±7.5 cm of their path, they share an aponeurosis. The belly of the ST usually spans only until the mid-thigh, after which its long tendon runs until its attachment in the upper part of the medial surface of the tibia, behind the attachment of the sartorius muscle and distal to the attachment of gracilis muscle. The terminal portion the ST tendon is united with the tendon of gracilis (further reinforcing the functional connections between the hamstrings and the adductor group), provides an expansion to the deep fascia of the leg and to the medial head of the gastrocnemius, and anatomically (and perhaps functionally) links the ST with the medial gastrocnemius. It is open to speculation whether pathology or dysfunction of the medial gastrocnemius interfere with the action of the ST. Usually, the midpoint of the ST receives a muscular slip from the BFlh, denoting a role in lateral force transfer.

Innervation to the ST is provided by the sciatic nerve (L5, S1, S2), through its tibial division. This pattern is shared with the SM, which lies deep to the ST, and by the BFlh, but not by the BFsh. Beyond the actions common to all hamstrings, the ST (as well as the SM) can medially rotate the knee when this joint is semi-flexed. With the hip extended, ST (and SM) can act to produce medial rotation of the thigh.

#### Semimembranosus

The SM also lies posteromedially in the thigh, deep to the ST. Proximally, it exhibits a long and flat tendon attached to the ischial tuberosity, and it receives fibrous expansions that flank the adductor magnus. Distally, the SM has a ramified pattern, sharing fibers with both ST and BFlh. Circa the mid-thigh, the SM gives off muscle fibers that converge to a second aponeurosis that attaches distally over the terminal tendon. The SM distal tendon divides into five components: anterior, direct, capsular, inferior and the oblique popliteal ligament (Beltran et al., 2003). The SM has a close anatomic relationship with the medial gastrocnemius, usually separated by a bursa.

Gray’s Anatomy describes that the SM can vary considerably in size and may be absent (which could potentially overload the ST) or double (which could potentially underload the ST). Proximally, the SM can arise from the sacrotuberous ligament instead of the ischial tuberosity. It can have muscular slips to the femur or to the adductor magnus. Although myotendinous and avulsion injuries are common in the SM, complete tears are seldom reported in the literature (Beltran et al., 2003).

#### Biceps Femoris

Unlike the ST and SM, the BF occupies a posterolateral position in the thigh. Proximally, the BFlh attaches to the ischial tuberosity, through a common tendon with the ST, but it can also insert into the sacrotuberous ligament. The BFlh has a fusiform belly, and its fibers terminate in an aponeurosis that also receives fibers from the BFsh. Pennation angle of the BFlh fibers is non-uniform, being greater in the proximal-middle sections, in comparison to the distal and extreme proximal sections (Huygaerts et al., 2021). The BFsh has its proximal attachment in the lateral lip of the *linea aspera* and this attachment may extend upward to almost the level of gluteus maximus. Distally, these muscles share a common tendon inserting into the head of the fibula and send expansions to the fibular collateral ligament and to the lateral condyle of the tibia. A variation noted in Gray’s Anatomy description is the absence of the BFsh.

While the BFlh shares innervation with the ST and SM, arising from the tibial division of the sciatic nerve (L5, S1, S2), the BFsh is innervated by nerves emerging from the same levels, but traveling in the common fibular division of the sciatic nerve. This differential innervation of the two heads of the BF may result in asynchrony and impaired coordination (Beltran et al., 2012; Huygaerts et al., 2021), potentially placing the BF at greater risk of injury (Burkett, 1975; Entwisle et al., 2017). Theoretical models have proposed that BF may be at increased injury risk in comparison with ST and SM (Dolman et al., 2014), owing to an additional BF compensation induced by a poor ST endurance, placing the BF at higher injury risk (Schuermans et al., 2014). In a retrospective study with 275 men soccer players who had sustained hamstrings injuries, the BFlh was the most commonly affected (56.5%), followed by the ST (24.4%), SM (13.7%), and BFsh (5.6%) (Crema et al., 2016). In Australian rules football players, injury of the BF represented 78% of all hamstrings injuries (Verrall et al., 2003). Beyond the actions common to all hamstrings, the BF can laterally rotate the knee when this joint is semi-flexed. With the hip extended, BF can act to produce lateral rotation of the thigh.

### Anatomic and Physiologic Variations of the Hamstrings

The hamstrings integrate the posterior compartment of the thigh, considered to be the most variable compartment (Tubbs et al., 2016). We explore known anatomic variations to unfold their implications for injury risk. It is unlikely that any single risk factor allows establishing a strong relationship with injury risk, but recognizing their existence may provide a more thorough understanding of individualized injury risk. Technological developments may translate that enhanced knowledge into practical applications. Table 1 summarizes the articles exploring anatomic variations of the hamstrings.

**TABLE 1:** Summary of studies cited in Section “Anatomic and Physiologic Variations of the Hamstrings.”

References	Population	Anatomic part assessed	Assessment method	Main findings
An et al. (2010)	50 lower extremity cadavers from 15 males and 12 females (age: 71 years)	Innervation patterns of HT	Dissection	BFsh and SM show one innervation entry, and BFlh and ST show two innervation entries. In those with two entries, for BFlh, the incidence of type I innervation was 82% (18% of type II) while for ST, the incidence of type I innervation was 14% (86% of type II).
Blazevich et al. (2006)	16 women and 15 men physically active (age: 19.9–20.6 years)	Architecture of quadriceps	Ultrasound	Description of the architecture of the quadriceps. Changes in the relative activation of individual muscles determines alterations in force, velocity movement range and contraction mode. Intramuscular activation changes with the movement requirements. Training should alter the pattern of activations to improve force transmission between muscles with different architecture, make these variations in activations efficient, and promote region-specific hypertrophic responses. There are little differences between sex in these adaptations.
Burkett (1975)	Review	Anatomical variation of HT	–	Extensive biceps femoris-femur attachment coupled with strength imbalances between HT increase the risk of strains.
Dahmane et al. (2006)	15 healthy sedentary males (age: 17–40 years) and 15 male sprinters (age: 23.2 ± 3.1 years)	Muscle fiber types of BF	Mechanomyography	Strong potential for the BF muscle to transform from slow to faster contracting muscle fibers after long-term sprint training.
Evangelidis et al. (2015)	30 healthy recreationally active individuals (age: 20.7 ± 2.6 years)	Aponeurosis of BFlh	MRI	The proximal aponeurosis size is highly variable between individuals, and it is not associated with to muscle size or knee flexor maximal isometric or eccentric strength. This disproportion may predispose those individuals with relatively small aponeurosis to hamstring strain injuries, as they could be subjected to greater mechanical strain in the muscle tissue surrounding the aponeurosis.
Evangelidis et al. (2017)	31 healthy, recreationally active participants (age: 21 ± 3 years)	Muscle fiber types of HT	MRI	HT muscles exhibited a balanced myosin heavy chain isoform distribution comparable to that of vastus lateralis, so that the predominance of fast-twitch fibers in HT muscle increasing the risk of strains is not supported.
Fiorentino and Blemker (2014)	12 male track and field athletes	Musculotendon of BFlh	MRI	A larger muscle and/or narrower proximal aponeurosis of the BFlh could predispose an athlete to an increased risk of injury by increasing peak local muscle tissue strain.
Fraser et al. (2013)	1 female cadaver (age: 87 years)	Anatomical variation of ST	Dissection	Two-part origin of the ST, with the variant portion being originated along the medial border of the ischial tuberosity. Could predispose to HT injury, chronic pain and pelvic floor discomfort.
Freitas et al. (2020)	40 male professional football players (age: 24.5 ± 4.9 years)	Aponeurosis of BFlh	MRI	There were no significant differences for size aponeurosis of the BFlh between players with and without previous BFlh injury.
Fridén et al. (1983)	12 male physical education students (age: 25 ± 7 years)	Muscle fiber types of vastus lateralis	Muscle biopsy	Type II fibers were more extensively damaged than type I fibers after 30 min of pedaling at a frequency of 60 rpm, at an intensity of 80–100% of VO_2_max, in a bicycle ergometer modified for use in eccentric work.
Galpin et al. (2012)	Description muscle fiber types	–	–	–
Garrett et al. (1984)	7 male and 3 female cadavers (age: 60 years)	Muscle fiber types of lower limb muscles	ATPase histochemical reaction	Relatively higher percentage of type II fibers in HT compared to other thigh and leg muscles.
Gérard et al. (2020)	SR: 10 RCTs evaluating 346 healthy adults (age: 18.3–29.6 years)	Architecture of BF	MRI and ultrasound	Eccentric strength training associated with increased fascicle length and muscle thickness, and decreased pennation angle, as well as eccentric strength of the HT.
Gray (1945)	2 male cadavers (age: 44 and 84 years)	Anatomical variation of HT	Dissection	All HT muscles originated from a common tendon in case 1. A muscle from the linea aspera and passing medially to the capsule of the knee joint, homologous with the ST, is presented in case 2.
Huygaerts et al. (2021)	Critical review	Muscle-tendon unit of HT	–	Fiber-fascicle lengthening is greater, and architectural structure non-uniform in the BFlh, with pennation being greater in the proximal-middle section compared to the distal and extreme proximal sections, that in addition to the inter-individual differences in BFlh structural features may predispose to this muscle to higher risk of HT injury.
Julien et al. (2018)	Review	Muscle fiber types	–	The changing environment could elucidate changes in muscle fiber characteristics, which could have implications in metabolism-related muscular atrophies.
Kellis (2018)	Narrative review	Architecture of HT	–	BFlh, compared to other HT, may be at higher increase of injury due to inter-muscular differences in HT architecture. Targeting the specific muscle-tendon region, instead of HT as a whole, could be beneficial in rehabilitation programs.
Kellis et al. (2009)	3 cadavers (age: 68.3 years)	Architectural parameters of BFlh and ST	Ultrasound dissection	High level of agreement in BFlh and ST architectural parameters measured through ultrasound compared to direct dissection.
Kellis et al. (2012)	8 cadavers (age: 67.8 years)	Architecture of HT	Dissection	The four hamstring components showed low to moderate architectural dissimilarity. Pennation angles were similar between BFlh, BFsh and SM, but higher than for ST.
Lievens et al. (2020)	32 male recreational athletes	Muscle fiber types of gastrocnemius	Magnetic resonance spectroscopy	Participants with predominantly fast typology fibers fatigues more markedly in repeated Wingate tests than participants with slow typology fibers.
Macaluso et al. (2012)	8 healthy, untrained individuals (age: 22 ± 1 years)	Muscle fiber types of vastus lateralis	Muscle biopsy	An acute bout of plyometric exercise preferentially affects type II fibers
Marušič et al. (2020)	40 healthy adults (age: 23.7 ± 2.5 years)	Architecture of BFlh	Ultrasound	A 6-week progressive eccentric HT training in a lengthened position showed positive effects for fascicle length (increase) and pennation angle (decrease), but not for muscle thickness.
Medeiros et al. (2020)	32 adult football players (age: 18–23 years)	Architecture of BFlh	Ultrasonography	An 8-week Nordic hamstring exercise training program was not effective at elucidating any improvement in muscle thickness, pennation angle, or fascicle length.
Mendiguchia et al. (2020)	32 adult football players	Architecture of BFlh	Ultrasound	The sprint training group showed moderate increase in fascicle length, and the Nordic group a small increase. The Nordic group presented a small increase at pennation angle.
Methenitis et al. (2019)	10 sedentary, 9 endurance runners, 10 power-trained and 9 strength-trained individuals	Muscle fiber composition of vastus lateralis	Muscle biopsy	Muscle fiber composition and rate of force development is affected by systematic training among different athletes. Type IIx fibers better correlated to rate of force development.
Rehorn and Blemker (2010)	1 healthy male individual	Aponeurosis of BFlh	MRI and 3D modeling	The fact that proximal aponeurosis is narrower than distal aponeurosis in BFlh could explain the prevalence of injuries near the proximal myotendinous junction in this muscle, and relative aponeurosis dimensions could also explain the more prevalence of injuries in BFlh compared to other HT muscles.
Schmuter et al. (2021)	1 female cadaver (age: 59 years)	Anatomical variations of BFlh and ST	Dissection	BFlh and ST were fused near their origin at the ischial tuberosity.
Shalabi et al. (2002)	14 males and 2 females (age: 26 years)	Muscle fiber types of ST	Muscle biopsy (percutaneous ultrasound-guided biopsy)	Patients following ACL reconstruction show a composition of 50 ± 13% type 1 fibers, 26 ± 8% type 2A, 23 ± 19% type 2B and 1 ± 1% type 2C. Muscle biopsy technique is adequate to identify muscle composition.
Solomon and Stevenson (2008)	1 male (age: 40 years)	Anatomical variation of BF tendon insertion	MRI	Case of a bilateral tibial insertion of the BF tendon in a previously asymptomatic patient, which could have implications on lateral knee stability.
Trappe et al. (2015)	1 world champion sprinter	Muscle fiber types of vastus lateralis	Muscle biopsy	Large proportion of vastus lateralis type IIx fibers in the sprinter, and power output from type IIa and IIx fibers was higher than any human values reported to date.
van der Made et al. (2015)	29 human cadaveric specimens (age: 71.5 years)	Anatomical variations of HT (origin dimensions, muscle length, tendon length, MTJ length and width, length of tendinous of ST (raphe).	Dissection	Overlapping proximal and distal tendons and muscle architecture may lead to a force not in line with the tendon and predispose to muscle injury. Protective effect of the presence of a raphe.
Vieira et al. (2007)	41 male and 56 female asymptomatic patients (age: 52.8 years)	Innervation patterns of BF	MRI	Description of the normal anatomy of the distal BF and the relationship with the peroneal nerve. The peroneal nerve can pass downward posterior to the BFsh and superficial to the lateral head of the gastrocnemius, but also in a tunnel between the two muscles (23%). An unusual relationship between the nerve and the distal BF could predispose to peroneal neuropathy.
Woodley and Mercer (2005)	3 female and 3 male cadavers (age: 69–88 years)	Architecture of HT	Dissection	ST, SM and BF showed anatomical partitioning defined by architecture and/or pattern of innervation. There was high degree of variation from subject to subject in most of the architectural features of the HT, such as, fascicular length, volume, physiological cross-sectional area or tendon characteristics.
Yang et al. (2018)	93 patients (age: 40.2 years)	Innervation patterns of BF	MRI	There were less participants with type I (38.7%) than type II innervation (61.3%), in which the thickness was lower. The course of common peroneal neuropathy through the “popliteal tunnel” formed between the BFsh and the lateral gastrocnemius muscle was approximately 40%.
Yasin et al. (2010)	25 patients undergoing ACL reconstruction with HT tendon autograph (age: 28 years)	Anatomy of accessory bands	Dissection	Gracilis and ST tendons showed a variable pattern of accessory bands, all of them occurring more than 11-cm proximal to the insertion of the tendons onto the tibial crest.

*HT, hamstrings; MTJ, musculotendinous junction; ST, semitendinosus; BFlh, biceps femoris long head; VO_2_max, maximal oxygen consumption; ATPase, adenosine triphosphatase; BF, biceps femoris; MRI, magnetic resonance imaging; ACL, anterior cruciate ligament; BFsh, biceps femoris short head; SM, semimembranosus; RCT, randomized controlled trial.*

#### Muscle Bellies

Cadaveric analysis showed that the extension of BFsh attachments to the femur vary substantially between individuals, leading to the hypothesis that individuals with more extensive attachments may apply more force, potentially increasing the risk of a strain injury (Burkett, 1975). The opposite interpretation is also possible, as attachments that are more extensive would allow the BFsh muscle belly to be more firmly attached to the femur, and therefore may be at less risk of a strain injury. The two heads of the BF have different proximal attachments and innervation, possibly representing muscles that were independent, but fused during the human evolutionary process (Tubbs et al., 2016). In some individuals the BFlh and BFsh remain separate, and occasionally the BFsh is absent (Tubbs et al., 2016). From 29 cadaveric specimens with a median age of 71.5 (range from 45 to 98), BFlh and BFsh extended approximately 42.0 and 29.8 cm, respectively (van der Made et al., 2015).

The ST can be partly fused with the SM (Tubbs et al., 2016) or the BFlh (Schmuter et al., 2021), and may present accessory slips to the coccyx, sacrotuberous ligament or to the ischial tuberosity (Fraser et al., 2013). Supernumerary ST bellies have been observed (Gray, 1945). Although ST and BFlh usually share a common proximal tendon attachment and aponeurosis, complete separation of these two muscles has been reported (Tubbs et al., 2016). Of note, the tendon of the ST may receive muscle slips from the quadratus femoris (Tubbs et al., 2016).

The SM can be doubled, absent, or split, and up to four bellies have been described inserting into the adductor magnus, planum popliteum and tibia (Tubbs et al., 2016). The ST, SM and BFlh may originate from a common tendon, which can be continuous with the intermuscular septum and, occasionally, envelop the piriformis muscle (Tubbs et al., 2016), providing another example of the connections between the hamstrings and other muscles of the lumbo-pelvic region.

#### Muscle Fiber Type

The muscle fibers vary in their morphology and histochemical properties (Galpin et al., 2012). Classifications of fiber muscle types have evolved through the years and certain classifications are still debated (Talbot and Maves, 2016). Here, we use the original classification reported by the authors being cited. There are important functional differences between different muscle fiber types, such as distinct metabolic properties (Mishra et al., 2015). There may be a bidirectional relationship, whereby metabolic environment also influences the characteristics of the muscle fibers (Julien et al., 2018). Type II muscle fibers are more easily fatigued than type I fibers (Lievens et al., 2020), and more susceptible to plyometric- (Macaluso et al., 2012) as well as eccentric-induced muscle damage (Fridén et al., 1983). Despite this information, eccentric actions have been emphasized by many programs for reducing injury risk of the hamstrings (van der Horst et al., 2015; Almeida et al., 2018; Elerian et al., 2019). Perhaps athletes with greater percentage of type II fibers should follow a more careful monitoring of the eccentric work they perform.

A previous cadaveric study with seven men and three women (37 to 76 years upon death) showed that type II fibers composed 48.5 to 59.5% of the proximal BFlh, 51.0 to 58% of the distal BFlh, 45.5 to 69.0% of the BFsh, 51.0 to 57.5% of the proximal ST, 50.0 to 69.5% of the distal ST, 47.5 to 54.5% of the proximal SM, and 44.0 to 55.5% of the distal ST (Garrett et al., 1984). In a study with 15 sedentary men aged 17 to 40 years old (Dahmane et al., 2006), the BF had a mixed fiber composition of ∼50% type 1 and ∼50% type 2 (25.2 ± 1.3% type 2a, 20.7 ± 1.4% type 2x and 5.7 ± 0.7% type 2c), meaning that BF has a considerable amount of fast-twitch fibers. Biopsies of the BFlh performed in 31 healthy, young men, showed a balanced distribution of myosin heavy chains (MHC), with 47.1 ± 9.1% MHC-I, 35.5 ± 8.5% MHC-IIA, and 17.4 ± 9.1% MHC-IIX (Evangelidis et al., 2017). For the ST, a composition of 50 ± 13% type 1 fibers, 26 ± 8% type 2A, 23 ± 19% type 2B and 1 ± 1% type 2C was found on biopsies from 16 patients 7–11 months after ACL surgery (Shalabi et al., 2002). In this reported ST composition (Shalabi et al., 2002), the standard-deviations of the reported ST muscle composition were relevant, especially in type 2B fibers, denoting interindividual variation.

It has been suggested that high-level sprinters are sharply different than the average person, with a percentage of up to ∼70% of type 2 fibers in the lower limb muscles, but this was derived from analysis of the vastus lateralis (Trappe et al., 2015), and should not be generalized to other lower limb muscles. Differences in fiber type composition of the lower limbs have been observed between endurance runners, power-trained individuals, and strength-trained individuals (Methenitis et al., 2019), but again these findings were extrapolated from observations of the vastus lateralis. *In vivo* knowledge of hamstrings muscle composition in humans remains largely unknown (Evangelidis et al., 2017).

As Lievens et al. (2020) pointed out, athletes with distinct muscle typology should train differently, and the individualization of training on the basis of this information is important to optimize performance and lowering the risk of potential injury. Unfortunately, knowledge of muscle fiber composition of the human hamstrings is scarce for building a broad picture. Furthermore, feasibility may require the development of more readily available, non-invasive, non-expensive technologies.

#### Pennation Angles

A study analyzed the pennation angles of BFlh and ST muscles from six legs derived from three male cadavers (Kellis et al., 2009). The BFlh and ST exhibited very similar pennation angles through cadaveric dissection (13.52 ± 2.35° versus 13.39 ± 3.31°) and ultrasound (13.88 ± 2.76° versus 13.34 ± 2.61°). The small standard deviation was suggestive of non-relevant intra- and interindividual variations. A posterior study of the same group dissected eight cadavers (age: 67.8 ± 4.3 years) (Kellis et al., 2012) and demonstrated that pennation angles of the BFlh, BFsh and SM were similar (13.46 ± 2.88°, 13.17 ± 2.60°, and 15.95 ± 2.39°, respectively), but substantially different from the pennation angle of the ST (9.14 ± 3.54°), which differs from the results found in their previous work (Kellis et al., 2009). The authors used a combination of mean fiber length, sarcomere length, physiological CSA, and pennation angle to calculate a similarity index (δ) between pairs of muscles, with lower values denoting greater similarity. While the BFlh and BFsh had a δ = 0.54, and BFlh and SM had a δ = 0.35, denoting a moderate similarity, there was low similarity between SM and ST (δ = 0.98) and between BFlh and ST (δ = 1.17). These findings underline that although the hamstrings are usually treated as a muscle group, there are relevant inter-muscular architectural differences between its individual muscles (Kellis et al., 2012).

A SRMA showed a limited to moderate confidence in evidence that eccentric training performed for a minimum of 4 weeks decreased the BFlh pennation angle (Gérard et al., 2020), which was also shown in a recent study (Marušič et al., 2020). However, another study with 32 soccer players (age, 18–23 years) performing an 8-week NHE program once a week versus twice a week showed no between-group differences in BFlh pennation angles (Medeiros et al., 2020). A prospective controlled study with soccer players analyzed the NHE and sprint interventions, showing that only the NHE induced small increases in the BFlh pennation angle (Mendiguchia et al., 2020), contradicting the decreases seen in previous studies (Gérard et al., 2020; Marušič et al., 2020). Results are conflicting, largely limited to the BFlh and involve a narrow set of exercise modalities. Further research is warranted to understand how different exercise modalities affect pennation angles.

#### Tendons

There is variation in the accessory bands of the hamstrings tendons (Yasin et al., 2010), as well as in tendon length. One study showed that the muscle belly of the SM originated at varying distances from the ischial tuberosity in different subjects, ranging from 8.6 to 14.5 cm (Woodley and Mercer, 2005). Interindividual variations in tendon-to-fiber length ratios may play a role in injury risk, as in the case of tendons of similar structure, tendon length critically influences compliance (Huygaerts et al., 2021). If two athletes have different ratios for the ST, it is possible that one will be at increased risk of injury than the other. It was hypothesized that bigger tendon-to-fiber length ratios provide a greater buffer to the muscle belly, potentially affording increased protection from injury, but this requires confirmation (Kellis, 2018; Huygaerts et al., 2021).

#### Aponeurosis

The aponeurosis may affect different muscle regions distinctly, demanding greater elongation of fibers in certain regions and less so in others (Blazevich et al., 2006; Huygaerts et al., 2021). A modeling study suggested that smaller ratios of aponeurosis to muscle width generated larger maximum peak local strain, postulating those larger muscles and/or narrower aponeurosis increased risk of injury (Fiorentino and Blemker, 2014). Interindividual variation in anatomic structure may put individuals with distinct aponeurosis to muscle width ratio at different levels of injury risk. However, an MRI study conducted in 30 healthy young men performing maximum voluntary actions of knee flexion showed interindividual variability in the area of the BFlh proximal aponeurosis, ranging from 7.5 to 33.5 cm (Evangelidis et al., 2015), and this area was not correlated with BFlh maximal anatomic CSA. The aponeurosis-to-muscle area ratio exhibited six-fold variability, with an interindividual ratio variation of 83%, and aponeurosis size was not related to isometric or eccentric knee flexion strength (Evangelidis et al., 2015). The authors stated that “individuals with a relatively small aponeurosis may be at increased risk of HSI” (Evangelidis et al., 2015, p. 1383), but this was not demonstrated.

A study using MRI to compare 80 thighs from 40 professional soccer players with (*n* = 9) or without previous (*n* = 71) BFlh injury in the preceding 3 years, suggested that proximal aponeurosis size of the BFlh was not an independent risk factor for HSI (Freitas et al., 2020). Notwithstanding, another computational modeling study suggested that varying the width, length and thickness of the BFlh aponeurosis had an impact on the location and magnitude of peak stretches within the muscle (Rehorn and Blemker, 2010). The authors found that location and magnitude of peak stretch could be explained by the difference in widths between the proximal and distal aponeurosis of the BFlh (Rehorn and Blemker, 2010). Aponeurosis characteristics may not represent an independent risk factor, but their interaction with factors such as tendon length, muscle belly anatomic CSA, among others, may provide further cues to better understand its relationship with hamstrings injury.

#### Innervation Patterns

Interindividual differences in innervation patterns of the hamstrings should be acknowledged, and their relationship with injury explored (Huygaerts et al., 2021). In some persons, the BFsh has two distinct regions, each innervated by a separate nerve (Woodley and Mercer, 2005). In the same study, two of the six subjects had a common trunk for nerve supply to SM and an inferior compartment for ST (Woodley and Mercer, 2005). The nerve to SM had branches to the adductor magnus muscle, highlighting the functional connections between the adductor magnus and the hamstrings. Woodley and Mercer (2005) showed that the primary branch of the nerve to SM, that supplied its distal region, had varied entry points (i.e., the location where the nerve branch pierces the muscle belly), from 22.5 cm distal to the ischial tuberosity in one, and 34.5 cm in another. Similar variations were found for both ST and BF (Woodley and Mercer, 2005). In a dissection of 50 cadaveric lower limbs, motor entry points and intramuscular nerve endings of the hamstrings were examined (An et al., 2010). In ST and BFlh, two distinct branching nerve patterns were found, classified into type I (only one primary motor branch emerging from the sciatic nerve) and type II (two primary motor branches) (An et al., 2010). In the BFlh, 82% of lower limbs presented a type I innervation, while in ST 86% had a type II innervation (An et al., 2010). Whether (and to what extent) subjects with different pattern type present alterations of motor coordination or how they affect functional performance is currently unknown.

In one study, two observers retrospectively reviewed one hundred 1.5-T knee MRI studies in 97 asymptomatic subjects (41 men and 56 women) for assessing anatomy of the distal BF (Vieira et al., 2007). The posterior extent of the BFsh was ≤1 cm in 50% of subjects, between 1–2 cm in 34%, between 2–3 cm in 15%, and ≥3 cm in one subject. The distal extent of the muscle belly of BFsh from the joint space varied from −2 cm in 5% of subjects to +2 cm in 2%, with 51% presenting 0 cm, and ∼42% showed +1 or −1 cm. The length and path of the BFsh showed interindividual variation in asymptomatic subjects. In this distal MRI assessment (Vieira et al., 2007), the muscle belly of the BFlh was identified in 40% of the subjects, and not visible in 60% of the subjects, suggesting that interindividual differences exist for muscle belly-to-distal tendon length ratios for the BFlh. In 77% of subjects the common peroneal nerve was situated superficial to the lateral head of the gastrocnemius and posterior to the BFsh; in the other 23% of subjects, a narrow tunnel between the lateral head of the gastrocnemius and the BFsh enveloped this nerve, but this tunneling effect did not result in neuropathy (Vieira et al., 2007).

A similar study retrospectively analyzed 1.5-T knee MRI scans of 93 Korean subjects, divided into types according to the course of the common peroneal nerve: type I (no tunnel) and type II (tunnel) (Yang et al., 2018): ∼40% of subjects were classified as type II, which is superior to the percentage observed in the aforementioned study (Vieira et al., 2007). This suggests that the prevalence of certain anatomic features may vary geographically (possibly related to genetic, environmental, and historical factors). In this study (Yang et al., 2018), type II subjects had significantly greater BFsh thickness. The functional relevance of these anatomic variations is not straightforward.

#### Attachment Sites

Despite traditional textbook description of the BF distal tendon as attaching to the fibular head (Standring, 2015), at least one case is described where the distal tendon of the BF inserted on the lateral aspect of the tibia, posterior to the iliotibial band and above the level of the fibular head (Solomon and Stevenson, 2008). In this case, the BF did have attachments to the fibular head, but these were muscular in nature. Whether this feature interferes with the mechanics of knee lateral rotation, implicates in the proximal actions (i.e., at hip joint level), or changes the coordination with other hip lateral rotators muscles (e.g., gluteus maximus, piriformis), remains speculative.

### Sex and Age-Related Differences in Hamstrings’ Anatomy and Physiology

This section addressed sex and age-related differences in hamstrings’ features, and Table 2 summarizes the articles consulted in this section.

**TABLE 2:** Summary of studies cited in Section “Sex- and Age-Related Differences in Hamstrings’ Anatomy and Physiology.”

Reference	Population	Anatomic part assessed	Assessment method	Main findings
Behan et al. (2018)	32 males (age: 20.6 years) and 34 females (age: 20.9 years) healthy, young, individuals with a low-moderate level of physical activity	Knee joint muscle morphology	MRI	Sex differences in muscle morphology that may predispose females to greater risk of ACL injury, primarily, a smaller knee flexors to knee extensors size ratio, but also a proportionately small sartorius and gracilis and a proportionately large vastus lateralis. Females have a larger BFlh as a proportion of the knee flexors than males, which may contribute to the higher risk of HSI in males.
Blackburn et al. (2009)	20 male (age: 20.7 years) and 20 females (age: 20.4 years) physically active individuals	Musculotendinous stiffness	Ultrasound	Musculotendinous stiffness was greater in males than in females, elastic modulus did not differ significantly across sex. Hamstring muscle size predicted 16% of the variance in hamstring musculotendinous stiffness.
Ebben et al. (2010)	12 male (age: 21.0) and 12 female (age: 19.9) university students	Magnitude and timing of hamstring activation, knee flexors-to-knee extensors ratio activation ratios, and knee flexors-to-knee extensors timing ratios of a variety of hamstring and quadriceps muscles.	EMG	In the precontact phase of jump landings and cutting: men and women are similar with respect to degree of activation of the hamstring In postcontact phase of the cut: men showed a trend toward higher knee flexors-to-knee extensors activation ratio than women
Hepple and Rice (2016)	Review	–	–	Aging conducts to changes in quantity and quality of motor unit, namely caused by motor neuron loss, neuromuscular joint instability, and repeating cycles of denervation and reinnervation leading to fiber type grouping.
Kirk et al. (2018)	11 healthy (age: 26) and 10 old (age: 80) male individuals		Intramuscular and surface EMG	Voluntary strength, evoked contractility, and MU discharge rates were diminished in old compared with young adult men No difference in relative surface EMG concurrent with significantly lower MU discharge rates may indicate that graded force generation in the hamstrings of old men is more dependent on MU recruitment. MU discharge rates of the SM and ST had a greater age-related effect compared to BF
Kong and Burns (2010)	25 males (age: 26.2) and 15 females (age: 24.2) healthy recreationally active individuals.	Hamstrings and quadriceps strength across multiple knee angles and angular velocities between the dominant and non-dominant legs	Isokinetic and isometric analysis (Biodex)	Knee flexors-to-knee extensors ratio was higher in the dominant leg than the non-dominant leg for both isometric and isokinetic measurements No difference in knee flexors-to-knee extensors ratio was found between males and females.
Lim et al. (2017)	10 young adults (24.2 ± 2.7)	Gluteus maximus, gluteus medius, vastus medialis, lateral hamstring, medial gastrocnemius, and soleus	Kinematic, force-plate and EMG	Increases in step length and frequency increases the contribution from the forces developed by gluteus maximus, gluteus medius, vastus medialis, medial gastrocnemius and soleus to both vertical support and forward progression. However, increase in step length results in greater differences in the contributions of vastus medialis and gluteus maximus and limb posture to vertical support.
Martín-San Agustín et al. (2019)	36 female (age: 21.1) and 34 male (age: 21.6) physically active individuals	Velocity contraction of BF and ST	TMG	Both male and female individuals had a similar pattern among the velocity of contraction
Narici et al. (2008)	Review	–	–	Training process apparently had no effect estimated relative length-tension properties of the muscle. Possibly, tendon stiffness and fascicle length increases canceled out each other.
Overend et al. (1992)	13 young (age: 24.5 years) and 12 old (age: 70.7 years) male individuals	Quadriceps and hamstring CSA	CT	Old male individuals had smaller quadriceps muscles and were weaker (22–32%) in knee flexion and knee extension at both angular velocities vs. young male Strength to CSA ratios were similar at 0 degree/s, but elderly had decreased ratios for both extensors and flexors at 120 degree/s. Correlations of knee extensor and flexor strength with muscle CSA were significant at both velocities in elderly men, but not at either velocity for the knee flexors in young men.
Roos et al. (1999)	13 young adults (26.2 ± 4.1) and 12 (80.0 ± 5.3) moderately active men	Quadriceps	Isometric dynamometer and EMG	Difference in age was observed on the voluntary and stimulated forces, while modest differences were found in contractible speed (slowest in older) and no change in the mean steady-state firing rates at any force level.
Ruas et al. (2019)	Review	Alternative methods of determining the knee flexors-to-knee extensors ratio as a measure of knee muscle strength balance.	–	There is not sufficient evidence to recommend any of the alternative methods of determining knee flexors-to-knee extensors ratio The higher reliability was found for rate for torque development knee flexors-to-knee extensors ratio
Smith et al. (2021)	125 football players	Association between hamstring strength, age and lower limb soft tissue injury history and subsequent hamstring injury		Increased age and previous hamstring, groin and calf injury are all associated with an elevated risk of subsequent hamstring injury in football players.
Vaughan et al. (2016)	Mice (11–13 months; and 15–21 months)	Extensor digitorum longus and soleus	Dissection	Significant increases of the number of Ia afferents in young compared to older mice. Fewer II afferents were also found in mice of middle and older age. However, intrafusal muscle fibers had no significant changes across the age. Thus, proprioceptive sensory neurons seem to degenerate prior to atrophy of intrafusal muscle fibers during aging.
Wan et al. (2017)	11 males (age: 23.6) and 10 females (age: 24.7 years) college students	Length, flexibility, and strength of hamstrings	3D modeling	Hamstring muscle optimal lengths were significantly correlated to hamstring flexibility score but not to hamstring strength The optimal knee flexion angle for maximal knee flexion moment decreased as hamstring flexibility score increased, which indicate that hamstring muscle optimal lengths may be affected by hamstring flexibility.
Webber et al. (2009)	Mathematical model	Quadriceps	Mathematical model	Changes in the Heckman-Binder motoneuron model for human data improved the frequency-current, and muscle unit force-frequency relationships. This adjustment resulted in lower firing frequencies in older and reduction in maximal force output.
Yoshiko et al. (2017)	15 young (21.0 ± 0.4 years old) and 15 old (70.7 ± 3.8)	Quadriceps femoris, hamstring and adductor	MRI	Age-related increase the intramuscular fat content, namely in the thigh areas, possibly explained by the loss of skeletal muscle cross sectional area in older.

*HT, hamstrings; ST, semitendinosus; BFlh, biceps femoris long head; BF, biceps femoris; MRI, magnetic resonance imaging; ACL, anterior cruciate ligament; BFsh, biceps femoris short head; SM, semimembranosus; TMG, tensiomyography; EMG, electromyography; CSA, cross-sectional area.*

#### Sex-Related Differences in Hamstrings’ Anatomy and Physiology

A study with recreational athletes (11 men and 10 women) assessed the relationship between hamstrings optimal length and hamstrings flexibility and isokinetic strength (Wan et al., 2017). While hamstrings muscle optimal length correlated with hamstrings flexibility, these factors did not demonstrate a relationship with hamstrings strength. For the same flexibility score, women had shorter hamstrings optimal muscle length than men. Thus, at any given ROM during a movement, hamstrings muscle maximal strain may differ between sexes. Another study used tensiomyography to assess normalized response velocity in BF, ST, rectus femoris, vastus medialis and vastus lateralis muscles of recreationally active young adult women (*n* = 36) and men (*n* = 34) (Martín-San Agustín et al., 2019). Comparisons between women and men were adjusted by height and mass. Sex-related differences were observed in velocity of action, with women having >15% differences between BF-to-quadriceps ratio, as well as ratios in the hamstrings, in comparison to men. These ratios should probably be termed knee flexors-to-knee extensors ratios, as more muscles than merely the hamstrings and the quadriceps are involved in the regulation of knee flexion and extension (Standring, 2015).

Sex-based differences in magnitude and timing of hamstrings and quadriceps activation during drop jump, sprint, and cutting (45°) tasks were assessed in 24 young adults (12 men, 12 women) (Ebben et al., 2010). In the post-contact phase of the cutting movement, men showed greater activation of all the hamstrings in comparison to women, while women produced longer bursts of the rectus femoris and vastus medialis activation. Hamstrings’ stiffness was shown to be greater in men than in women, but without differences in stress, strain, and elastic modulus (Blackburn et al., 2009). Blackburn et al. (2009) speculated that the smaller hamstrings stiffness observed in women could compromise their ability to resist changes in length associated with joint perturbation. However, the authors recognized that the sex-related differences in hamstrings stiffness could be partly attributed to anatomic CSA (Blackburn et al., 2009). It has been suggested that age or training level are more relevant than sex to explain differences in knee flexors-to-knee extensor ratios (Kong and Burns, 2010). It is also possible that the specific method used to determine knee flexors-to-knee extensors ratios (e.g., ratios calculated by angle-specific torque, rate of torque development) provide different results [for more information, see Ruas et al. (2019)].

Other evidence suggests that sex-related anatomic differences play a prominent role in explaining the differences in knee flexors-to-knee extensors ratios between men and women. A study with 1.5T MRI of healthy, but untrained young men (*n* = 32) and women (*n* = 34), showed sex-related differences in the maximal anatomic CSA of knee flexors and extensors (Behan et al., 2018). Women had a smaller ratio of knee flexors to knee extensor anatomic CSA. Although the hamstrings are not the only knee flexors, they play a prominent role in this action. In comparison with men, women showed a greater proportion of vastus lateralis, BFlh and SM in relation to their respective muscle groups. Conversely, women showed a lesser proportion in sartorius, gracilis and BFsh.

#### Effects of Aging in Hamstrings’ Structure and Function

There is limited evidence regarding the effect of aging in hamstrings’ anatomy, although ages >25 years have been associated with increased risk of hamstrings injury (Smith et al., 2021). Kirk et al. (2018) found that voluntary strength, evoked contractility, and motor unit (MU) discharge rates were diminished in a group of 10 elderly (mean age: 80 ± 5 years) compared to a group of 11 young men (26 ± 4 years). These findings could be due to the infiltration of non-contractile tissue in aged hamstrings (Overend et al., 1992; Yoshiko et al., 2017), altered MU facilitation, age-related remodeling (Webber et al., 2009; Hepple and Rice, 2016), muscle fiber membrane dysfunction, decreased sarcoplasmic reticulum function, altered calcium ion kinetics, and changes in connective tissue elements (Narici et al., 2008; Hepple and Rice, 2016). Hamstrings have a significantly lower MU discharge rates in the elderly, as opposite to findings in the vastus medialis portion of the quadriceps (Roos et al., 1999). *In vitro* and animal studies suggest that the loss of proprioceptive sensory neurons and innervation differences occur at different rates between flexors and extensors during the aging process (Vaughan et al., 2016).

Additionally, ST and SM could be more affected by aging than BF, providing evidence that musculature and neuromuscular system could be differently impacted by aging (Kirk et al., 2018). As a longer step length may require higher contributions from the hip and knee extensor musculature (Lim et al., 2017), hamstrings modifications with aging, and the decreased muscle strength, may explain why elderly people choose shorter length steps and walk more slowly (Lim et al., 2017). These findings warrant further investigation.

### The Consequences of Previous Knee Surgeries

Knee surgeries often harvest hamstrings tendons, potentially increasing the risk for future hamstrings injuries. When harvesting hamstrings tendons for surgical purpose, such as patellar tendon (Friedman et al., 2020) or ACL reconstruction (Keyhani et al., 2020), iatrogenic nerve injuries can occur (Colombet and Graveleau, 2016; Ruffilli et al., 2017). For example, the common fibular nerve emerges posterior to the BF distal tendon, to which it adheres (Standring, 2015); collecting part of the BF tendon for knee surgeries presents considerable risks. Consequently, the ST has been a common choice (comparable to gracilis in terms of effects) for transfer in surgical treatments (Yasin et al., 2010; Colombet and Graveleau, 2016), including those for the ACL (Horteur et al., 2020; Keyhani et al., 2020), medial collateral ligament (Cao et al., 2016) and patellar tendon (Jain et al., 2014; Friedman et al., 2020).

In a study of female soccer players (*n* = 90) that had undergone ACL reconstruction, with data available for a minimum of a 2-year follow-up, the outcomes for hamstrings autograft and bone-patellar tendon-bone autografts were very similar (Britt et al., 2020). Notwithstanding, a meta-analysis of 5,561 patients undergoing ACL reconstruction found that hamstrings autografts were less likely than bone-patellar tendon-bone autografts to incur in a contralateral ACL rupture (Zhou et al., 2020). The safety of surgical procedures and its effects on the specific injury addressed should not be disregarded (Jain et al., 2014). Nonetheless, these autografts may increase the fragility of the hamstrings and put them at increased risk of sport-related injuries. One study assessed recreationally active participants (five men and nine women) that had returned to sport after unilateral ACL reconstruction using ST tendon autografts (Messer et al., 2020). The athletes had undergone surgery between 12 to 78 months prior to the study and MRI was used to compare the surgical limb to the other limb. The surgically treated limbs’ STs had significantly smaller anatomic CSA and muscle volume than non-surgical limbs, and the surgically treated limbs also exhibited a lower exercise-induced transverse relaxation time. Surgically treated limbs also exhibited *higher* volumes of the SM and BFsh, perhaps to compensate for the lower volumes of the ST (Messer et al., 2020).

Using ST autografts for ACL reconstruction has long-term consequences for the hamstrings of the surgically treated limb, such as ST and gracilis hypotrophy (Sherman et al., 2021). Although alternatives such as peroneus longus autografts are being explored for ACL reconstruction (Rhatomy et al., 2019), it is possible that could bring some problems for the ankle and foot, since peroneus longus (a.k.a., fibularis longus) acts in eversion and plantar flexion of the foot and also provides support to the longitudinal and transverse arches of the foot (Standring, 2015). Trade-offs are unavoidable, and their consequences should be acknowledged and addressed by rehabilitation programs, requiring multifactorial approaches including different modalities of strength training, variation in the exercises, balance knee- and hip-dominant exercises, and careful managing of loading (Buckthorpe et al., 2020).

Albeit inadvertently, surgical procedures that extract tissue from the ST may compromise the endurance of this muscle (Vairo, 2014; Lee and Lee, 2020). Consequently, the BF will produce greater force and for a longer period to compensate for this reduced capacity of the ST (Schuermans et al., 2014). This could lead to the BF fatiguing earlier and/or being exposed to loads that exceed its capacity. Paradoxically, this may expose the “healthier” muscle (i.e., BF) to increased injury risk, and may explain why the majority of acute HSI occur in the BFlh (Freitas et al., 2021; Huygaerts et al., 2021). Still, a study with healthy subjects performing an indoor running task showed that the largest peak strain was achieved by the BF, while the highest peak force and the most power and work were generated by the SM (Schache et al., 2012), so this issue is open to debate.

### Synopsis of the Second Part

The hamstrings complex comprises the posterior compartment of the thigh, consisting of the ST, SM, and BF (BFlh and BFsh) muscles. They are biarticular muscles that act as hip extensors and knee flexors and rotators and play a critical role in several daily and sports activities. Regarding the main hamstrings anatomic and physiological variations discussed in the previous sections, it was shown that a high degree of variability exists in many of the architecture variables addressed. Lower tendons and aponeurosis to muscle ratios, higher number of type II fibers, variations in attachment sites and innervation patterns, and knee surgeries in which portions of the ST are extracted, could place the hamstrings complex musculature, especially the BF muscle, at increased risk of injury. Additionally, neuromuscular variations in women and the aging process affect the hamstrings musculature, further increasing the risk of hamstrings injury in women and elderly people.

The high level of anatomic variability from subject to subject and the variability of the study designs used in the literature, makes it difficult to draw general recommendations, and advises against adopting one-size-fits-all exercise programs. Since most sports-related injuries have a clear multifactorial nature (Monajati et al., 2016; Green et al., 2020; Lahti et al., 2020), studies may fail to find a clear relationship between the injury and any given risk factor. This does not imply that a particular risk factor is irrelevant in the probability of suffering a hamstrings injury. Although the study of resting muscle architecture of the hamstrings may provide relevant insights, it is unclear whether this provides valid information for dynamic architecture and gearing during dynamic movements (Huygaerts et al., 2021).

## Concluding Remarks

**FIGURE 2:**
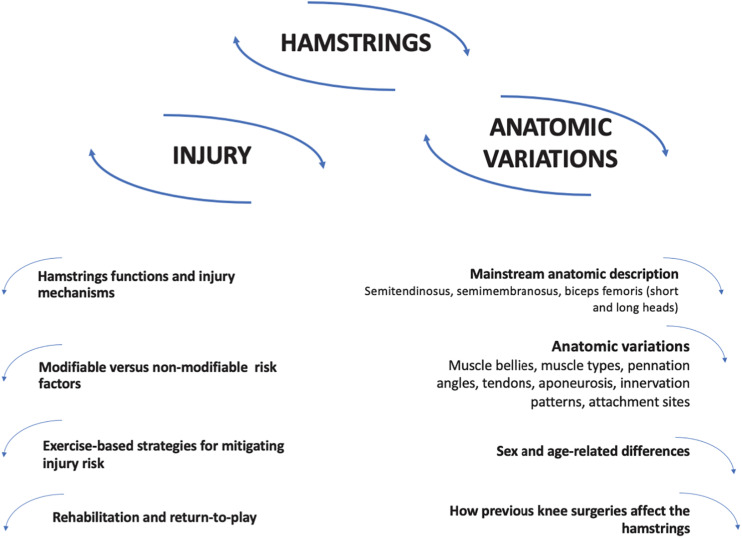
Overview of concepts and hamstrings anatomic and physiologic variations.

Hamstrings injuries present a challenge in sports science and practice, and many exercise-based programs have been proposed to mitigate injury risk, focusing on modifiable injury risk factors. But scientifically reported data shows that non-modifiable risk factors potentially play a more relevant role than modifiable risk factors. This suggests that greater efforts should be implemented to understand and identify the non-modifiable risk factors, such as relevant anatomic variations (e.g., tendons, aponeurosis, fiber types). The possession of this enhanced knowledge may help designing better-individualized exercise interventions, considering the non-modifiable particularities of the athlete. If a certain anatomic variation puts the athlete at greater risk of injury (e.g., nerve entrapments or tunneling), training programs can design better-individualized stimuli instead of delivering one-size-fits-all solutions. The recognition and assessment of non-modifiable risk factors allows coaches to act upon them, i.e., to prescribe exercise programs that are well-suited for those identified variations. As an example: if athletes with a greater percentage of type II fibers incur in greater damage induced by eccentric exercise, coaches can reduce the weekly volume, intensity and/or frequency of eccentrically biased exercises. Figure 2 synthesizes the concepts and domains approached in our work.

The literature has identified the need for prospective studies evaluating the influence or architectural variables in hamstrings injury, as well as the importance of targeting architectural changes in injury reduction programs, highlighting that ultrasound is potentially the most reliable technique to detect such changes (Behan et al., 2019). In the case of non-modifiable anatomic variations (e.g., number of muscular insertions into bone), it would be important to explore differential responses to exercise protocols, providing coaches with the tools for better individualizing training prescription. Of course, it is quite complicated to carry out the “gold-standard” study designs required to detect risk factors and validate screening tools (Bahr, 2016), as large-scale prospective, long-term, randomized studies assessing multiple outcomes at multiple time points are very difficult to implement. Still, this should not discourage researchers from progressively better and more complete attempts to address this complex, yet fascinating topic. We therefore encourage researchers to develop more large-scale prospective studies, including some of the mentioned architecture variables, with reliable yet non-invasive techniques such as ultrasound, to widen the body of knowledge regarding this subject matter.

As limitations of this work, we did not perform a systematic review of the literature, given the scope and goals. It is possible that relevant information has eluded our searches, although we tried our best to provide and thorough and balanced account. Where controversies existed, we attempted to explore its complexities and provide contradictory accounts.

## Practical Implications

Improved knowledge of interindividual variations in hamstrings anatomy and physiology will help coaches designing better individualized exercise programs. As an example, athletes with greater percentage of type II muscle fibers in the hamstrings should probably be allowed greater recovery times between sessions of eccentrically biased exercise. Muscle bellies with more extensive attachments to the femur may provide some degree of injury protection when producing forces of great magnitude, but this requires further exploration. Pennation angles, tendon-to-fiber length ratios, attachment sites and features of the aponeurosis, as well as the interaction between these factors, have unclear relationships with injury risk, with further research required before practical implications are promoted. The extensive anatomic and neurologic connections between the hamstrings and the gluteal and adductor muscles suggests that training programs should include exercises that demand the interaction of these three muscle groups, instead of relying solely on more hamstring-dominant exercises (e.g., the NHE).

While coaches tend to focus on modifiable risk factors, such as strength and endurance, non-modifiable risk factors may be acted upon, by designing exercise interventions that better comply with the identified characteristics. For example, in the case of athletes with previous knee injuries using ST autografts (non-modifiable factor), coaches should recognize that hamstrings endurance may be compromised, designing exercise interventions that gradually improve their endurance, but also recognizing that those athletes may benefit from shorter training sessions and/or shorter durations of play in matches. Improvements in the quality, availability and costs of imaging techniques will expand the assessment of interindividual anatomic variations, better individualizing exercise prescription.

## Author Contributions

JA, SR-R, and FMC contributed to conception and design of the review. MA, PN, HS, AF, JO-J, and RR-C wrote sections of the manuscript. All authors contributed to manuscript revision, read, and approved the submitted version.

## Conflict of Interest

The authors declare that the research was conducted in the absence of any commercial or financial relationships that could be construed as a potential conflict of interest.
